# The Healthy and Diseased Equine Endometrium: A Review of Morphological Features and Molecular Analyses

**DOI:** 10.3390/ani10040625

**Published:** 2020-04-05

**Authors:** Sandra Schöniger, Heinz-Adolf Schoon

**Affiliations:** 1 Targos Molecular Pathology GmbH, Germaniastrasse 7, 34119 Kassel, Germany; 2 Institute of Veterinary Pathology, Faculty of Veterinary Medicine, Leipzig University, An den Tierkliniken 33, 04103 Leipzig, Germany; schoon@vetmed.uni-leipzig.de

**Keywords:** biomarker, endometrial diseases, equine, mare, molecular mechanisms, pathology, physiology, review

## Abstract

**Simple Summary:**

Diseases of the endometrium are a frequent cause of subfertility in mares and have an economic impact on the horse breeding industry. These include periglandular fibrosis of endometrial glands (endometrosis), degenerative diseases of vessels (angiosis), inflammation (endometritis), as well as altered differentiation of endometrial glands. Some mares are susceptible towards persistent endometritis. The etiology and pathogenesis of endometrosis are still unclear. This review describes morphological hallmarks and molecular features associated with endometrial health and different types of diseases. The presented literature data reveal characteristic differences in the expression of several extra- and intracellular molecules between the healthy and diseased equine endometrium. Some of these molecules can be detected directly within the tissue and thus have the potential to serve as excellent diagnostic markers for the presence of endometrial diseases. The knowledge of disease-associated changes in cellular differentiation, secretory functions, and immune mechanisms will help to decipher pathogenesis and will contribute to the development of novel treatments. In addition, the quantification of molecular alterations may contribute to a fertility prognosis for an individual mare. Reproductive health increases the well-being of mares and reduces financial loss for the horse breeding industry.

**Abstract:**

Mares are seasonally polyestric. The breeding season in spring and summer and the winter anestrus are flanked by transitional periods. Endometrial diseases are a frequent cause of subfertility and have an economic impact on the horse breeding industry. They include different forms of endometrosis, endometritis, glandular maldifferentiation, and angiosis. Except for suppurative endometritis, these are subclinical and can only be diagnosed by the microscopic examination of an endometrial biopsy. Endometrosis is characterized by periglandular fibrosis and nonsuppurative endometritis by stromal infiltration with lymphocytes and plasma cells. The pathogenesis of endometrosis and nonsuppurative endometritis is still undetermined. Some mares are predisposed to persistent endometritis; this has likely a multifactorial etiology. Glandular differentiation has to be interpreted under consideration of the season. The presence of endometrial diseases is associated with alterations in the expression of several intra- and extracellular molecular markers. Some of them may have potential to be used as diagnostic biomarkers for equine endometrial health and disease. The aim of this review is to provide an overview on pathomorphological findings of equine endometrial diseases, to outline data on analyses of cellular and molecular mechanisms, and to discuss the impact of these data on reproduction and treatment.

## 1. Introduction

Endometrial diseases of mares are an important cause of subfertility. These include endometrosis, endometritis, glandular differentiation disorders, and angiosis (synonym: angiosclerosis) [1,2,3,4,5,6,7,8,9]. Individual mares often display the concurrent presence of several endometrial diseases [1,3]. Transient breeding-induced endometritis with a duration of less than 72 h is a physiological reaction [3,10,11]. Some mares, however, are predisposed to persistent endometritis (“susceptible mares”) that often develops after breeding [2,11,12]. 

Except for suppurative endometritis, endometrial diseases can only be revealed by the histopathological examination of an endometrial biopsy [1,3]. Importantly, the endometrial biopsy of a mare should be interpreted under consideration of signalment, clinical history, and season of the year [3,7,9]. Fertility-influencing factors include the age of the mare [7], the length of barrenness [1,7], and a previous long-term use of the mare in athletic performances [13]. 

The pathogenesis of nonsuppurative endometritis and endometrosis is unknown, and no routinely available treatment exists [2,8,14]. Moreover, nonsuppurative endometritis often persists despite treatment [3,12].

In recent years, a major research focus in equine endometrial pathology was to reveal disease-associated cellular and molecular mechanisms [15,16,17,18,19,20,21,22,23,24,25,26,27,28]. This will help to gain further insights into the pathogenesis of endometrial diseases [15,16,17,18,19,20,21,22,23,24,25,26,27,28], and it may contribute to the development of effective prophylactic regimes and novel treatment options. Some of the identified biological parameters have the potential to be used as biomarkers for endometrial health and disease. 

A biomarker is a biological indicator of a pathophysiological process or of a treatment response that can be objectively evaluated, e.g., an enzyme as a marker of cellular activity or cytokines as markers of the type of inflammation [29,30,31]. In addition to their use for the diagnosis of a pathological condition, some biomarkers have prognostic value or can predict the response to a certain treatment [29,30,31].

The aim of this review is to provide an overview about endometrial diseases of mares from a pathologist’s perspective with emphasis on morphological hallmarks and associated cellular and molecular mechanisms. In addition to providing insights into the diagnostic work-up and pathophysiology of these diseases, it is also aimed at assisting research into novel prophylactic regimens as well as new treatment options. 

## 2. Physiological Characteristics of the Equine Endometrium

### 2.1. Endometrial Histology and Influencing Factors

**Figure 1:**
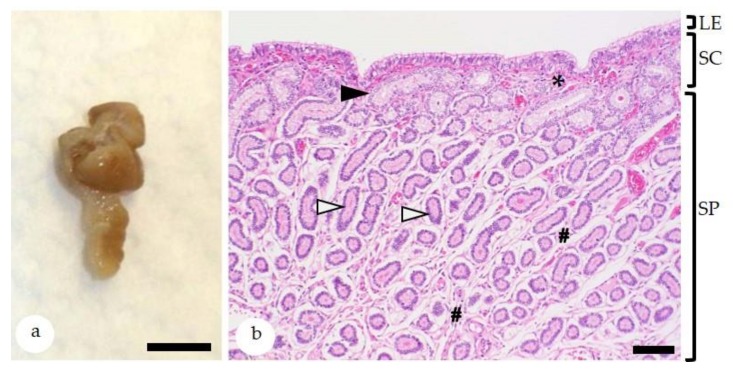
(**a**) Equine endometrial biopsy. Bar = 0,5 cm; (**b**) histologically, the endometrial biopsy sample is composed of the luminal epithelium (LE) and the lamina propria mucosa with the superficial stratum compactum (SC) and the deeper stratum spongiosum (SP). The stratum compactum has a dense stroma (asterisk) and contains the glandular ducts (black arrowhead). The stratum spongiosum has a loosely arranged stroma (hash keys) that surrounds the secretory portions of the endometrial glands (white arrowheads). Bar = 200 µm; Staining with hematoxylin-eosin.

**Figure 2:**
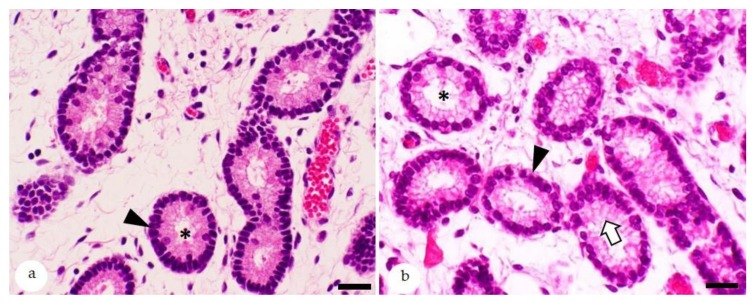
Histological features of equine endometrium stained with hematoxylin-eosin; (**a**) estrus: glands with a proliferative morphology. These are lined by epithelia with ovoid to elongate nuclei (arrowhead) and have narrow lumina (asterisk); (**b**) mid-diestrus: secretory differentiation of endometrial glands. Lining epithelial cells have round basally located nuclei (arrowhead) and apical foamy cytoplasm (white arrow) and wide glandular lumina (asterisk). Bars = 30 µm.

The histological features of the equine endometrium [32,33,34] are depicted in Figure 1. In the Northern hemisphere, the winter anestrus and the physiological breeding season in late spring and summer are flanked by the autumn and spring transitional periods (ATP and STP), respectively [35]. The precise duration of these periods in an individual mare is difficult to predict due to the influence of several factors including environmental conditions (e.g., temperature and lighting) as well as nutrition and body weight of the mare [35]. The endometrial biopsy serves as a sensitive indicator for the ovarian function of a mare [3]. Endometrial biopsies collected during the winter anestrus display seasonal endometrial atrophy [2]. During the physiological breeding season, ovarian cycles are regular and endometrial glands are active [35]; functional endometrial morphology (proliferative and secretory) is consistent with the follicular and luteal phases of the estrous cycle, respectively (Figure 2) [2]. The endometrial cycle of the mare has a duration of 21–22 days; day 0 is defined as ovulation day [35,36]. During the proestrus (days 18–20; synonym: preestrus), estrus (days 21–1) and postestrus (days 2–4; synonym: metestrus) endometrial glands show a proliferative differentiation, whereas during the early and mid-diestrus (days 5–13), a secretory differentiation is observed (Figure 2) [2,33,36]. During the late diestrus (days 18–20), glandular involution takes place [2,36]. During proestrus, stromal edema induces nesting of individual gland branches that need to be distinguished from periglandular fibrosis [2,33,36]. The density of glands per unit of endometrial area is less during the proliferative than during the secretory phase of the endometrial cycle. The diestrus-associated higher glandular density is caused by increased glandular tortuosity and reduced stromal edema [2,33,36]. In comparison, the proliferative phase is characterized by straightening of glands and prominent stromal edema [2,33,36]. Since the biopsy procedure can also evoke stromal edema [33], morphological features of glandular epithelia are very helpful for determination of the stage of the endometrial cycle. Glands with a proliferative differentiation are lined by columnar epithelial cells with eosinophilic cytoplasm and basally located ovoid to elongate nuclei. Their lumina are narrow to medium-sized [6,36,37,38]. During postestrus, the transition from a proliferative to a secretory morphology is evidenced by pale staining of the cytoplasm, reduced density of nuclear chromatin, and an increase in luminal size [6,38]. Glands with a secretory differentiation have columnar epithelial cells with basal round to ovoid hypochromatic nuclei and vacuolated apical cytoplasm. Their wide lumina may contain secretory material [6,36,37,38]. 

The activity of the hypothalamic–pituitary gland–ovarian axis gradually increases during STP and decreases during ATP [35]. Accordingly, biopsies collected during early, mid-, and late STP show a gradual increase in glandular activity [9], whereas those obtained during early, mid-, and late ATP display a successive decrease in glandular activity [9]. An irregular differentiation during the transitional periods is observed in 22–30% of the biopsies and is regarded as a physiological finding likely evoked by irregular ovarian cycles [9].

### 2.2. Expression of Estrogen and Progesterone Receptors during the Estrous Cycle 

By immunohistochemistry, estrogen receptors (ESRα and ESRβ) and progesterone receptors (PGR) are detected in luminal epithelial (LE) and glandular epithelial (GE) cells and stromal cells [36,39,40,41,42]. Most studies observe a solely nuclear immunostaining [36,39,40,41], whereas Silva et al. [42] report nuclear and cytoplasmic reactions for ESRα in LE and GE cells and for ESRβ in GE cells. Biopsies collected during estrus (days 20 and 0 of the cycle) show a higher expression of ESRα in LE and GE cells, and stromal cells than those obtained from mares in mid- and late diestrus (days 9 to 14 of the cycle) [41,42]. Similar variations in hormone receptor expression between estrus and mid- to late diestrus are also detected for PGR [41] but not for ESRβ [42]. 

The study of Aupperle et al. [40] examines the cycle and cell-type-dependent variations in immunostaining for ESR and PGR in endometrial biopsies collected repeatedly over the course of the same estrous cycle (days 0, 5, 10, 13, 16, and 19) from each of 7 mares. Although this study refers to ESR, it appears likely that ESRα was examined due to the detected differences in the hormone receptor expression between estrus and mid- and late diestrus and the common synonymous use of the designations ESR and ESRα [43]. The highest levels of ESR and PGR are expressed in epithelial cells of endometrial glands at the early diestrus (day 5 of the cycle), whereas at this time, stromal cells show the lowest immunostaining for these receptors [40]. After that, the protein expression of ESR and PGR in GE cells decreases and reaches lowest levels in mid-diestrus (day 13) [40]. This is followed by their gradual increase in GE cells [40]. In comparison, stromal cells display an increasing staining for ESR and PGR throughout the diestrus, and highest values are present at the ovulation day (day 0) [40]. Staining of ESR and PGR in luminal epithelia and glandular ducts is relatively low during the entire estrous cycle [40]. The detected cycle-dependent expression of ESRα and PGR indicates a stimulatory effect of estradiol and an inhibitory impact of progesterone on the protein expression of ESRα and PGR [40,41,42].

Data obtained from mouse models [44,45,46] as well as mouse [47] and human endometrial cell cultures [48] show that sex hormone-induced morphological and functional changes during the endometrial cycle are dependent on an intimate crosstalk between epithelial and stromal cells mediated by paracrine factors [44,45,46,47,48]. These bind to their receptors on the target cell population followed by induction of signal transduction and changes in cellular functional activity [47]. In this regard, mitotic activity of endometrial epithelial cells is stimulated by binding of estradiol to ESRα on stromal cells [44,46,47,48], and insulin growth factor I [48] as well as Bmp8a and Fgf10 have been identified as stromal derived paracrine mitogenic factors [47]. Ligation of progesterone to PGR on stromal cells antagonizes estrogen-induced epithelial proliferation [46]. Estrogen-mediated epithelial secretion of lactoferrin requires expression of ESRα on both cell populations, i.e., epithelial and stromal cells [45,46]; its inhibition by progesterone is also dependent on PGR expression on epithelial and stromal cells [45].

### 2.3. Expression of Glandular Secretory Proteins

A high expression of uterocalin [49,50,51], calbindin_D9K_ [51], and glycogen [51] is mainly associated with high progesterone levels. Since uterocalin is a lipocalin of 19 kDa, it had been initially designated as P19 [52]. In detail, immunostaining for uterocalin in GE cells is undetectable [49] to mainly low [51] during estrus, weak at day 2 of the cycle GE cells [49], and strong in association with high serum progesterone levels [49,51]. A higher expression and activity of the enzyme hexokinase 2 that is involved in glycogen synthesis occurs in parallel to the increased glycogen detection in GE cells during diestrus [53]. In contrast, maximal immunostaining for uteroglobin and uteroferrin is mainly observed after a decrease in serum progesterone [51]. Individual variations between mares exist [51]. 

### 2.4. Expression of Cytoskeletal Proteins

In non-diseased endometria, GE cells are diffusely pancytokeratin immunopositive [15,28], negative for smooth muscle actin (SMA) [27,28], almost always vimentin-negative [15,27,28], and occasionally calponin-positive [28]. Examining biopsies of non-diseased endometria from 10 mares, Minkwitz et al. [28] report a mean value (MV) of 1.7% and a standard deviation (SD) of 2.8% for glands that show calponin expression. 

In endometria with endometrosis, healthy glands are described as vimentin-negative by Aupperle et al. [15], whereas Minkwitz et al. [28] detect very few healthy glands with epithelial vimentin immunoreaction (MV ± SD: 0.1 ± 0.3). Bischofberger et al. [27] examine the percentage of positive GE cells in healthy glands from endometrial biopsies with unequal glandular differentiation (UGD) or mild endometrosis and diagnose up to 10% vimentin-positive GE cells. Healthy glands from endometria with endometrosis seldom show desmin expression (MV: 0.8%; SD: 3.4%) [28]. Calponin immunostaining also occurs in a small number of healthy glands from endometria with endometrosis [28]. The percentages of glands with calponin expression slightly increases in endometria with rising degrees of endometrosis, i.e., MVs of 2.6%, 3.2%, and 11.6% are reported in endometria with mild, moderate, and marked endometrosis, respectively [28]. 

Stromal cells from healthy endometrial areas are variably vimentin-positive [15,27,28]. Bischofberger et al. [27] report that percentages of vimentin-positive cells vary between less than 10% and 100% [27]; Minkwitz et al. [28] provide an MV of 31.3% and an SD of 24.9%. In addition, small numbers of stromal cells from healthy areas are desmin-positive [27,28]. Bischofberger et al. [27] detect mainly between 1% and 20% desmin-positive stromal cells and Minkwitz et al. [28] provide an MV of 11.1% and an SD of 12.8%. SMA expression is observed only in a small number of stromal cells (MV: 0.6; SD: 1.0) by Minkwitz et al. [28], whereas Bischofberger et al. [27] diagnose up to 20% SMA-positive stromal cells. Moderate numbers of stromal cells (MV: 15.8; SD: 19.5) from healthy areas are calponin-positive [28]. There are no marked differences between the stromal expression of vimentin, desmin, SMA, and calponin in the non-diseased endometrium [27,28] as well as healthy areas of biopsies with endometrosis [27,38] or UGD [27].

### 2.5. Resident Immune Cells 

Scattered neutrophils can be observed within the stratum compactum (SC) and luminal epithelium only during estrus but not during the other stages of the endometrial cycle [2,36]. Throughout the endometrial cycle, a few eosinophils are a physiological finding [2,36,54]. The SC contains slightly higher numbers than the stratum spongiosum (SP) [54]. Grimm et al. [54] report the numbers of eosinophils in endometria of 10 mares per 40× high power field (HPF); eosinophil counts range from 0 to 11 in the SC and from 0 to 5 in the SP. The highest values are observed during mid-diestrus (day 13) [54]. Although mast cells slightly increase at estrus, no statistically significant differences in the numbers of mast cells exist over the estrous cycle [55]. Higher numbers of mast cells are present in the SP than the SC [54,55]. By enumeration of mast cell numbers in 15 adjacent 9,483.494 µm^2^ fields of view, mean mast cell counts in the SC and SP are 4.5 and 16.5, respectively [55]. Notably, mast cells in the equine endometrium are pleomorphic with round, ovoid, or elongate shapes [55].

Low numbers of resident CD3+ T lymphocytes are present within the luminal and glandular epithelium as well as the stroma of the SC and SP [18,22]. CD3+ cells are more numerous in the SC than in the SP [22]. According to Rudolph et al. [22], CD3+ T lymphocytes in the SC and SP range from 74 to 96 and from 21 to 44 per five 40× HPFs, respectively. Numbers of CD3+ T lymphocytes in the luminal and glandular epithelium vary from 4 to 17 and from 5 to 26 per five 40× HPFs, respectively [22]. The SC contains also more resident CD4+ lymphocytes and CD8+ lymphocytes than the SP [22,56]. By calculating the mean value of CD4+ and CD8+ in endometrial biopsies with categories I or IIa [1], Watson and Thomson [56] report slightly higher numbers of CD4+ cells in the SC and approximately equal numbers of CD4+ and CD8 + cells in the SP. Although the investigation of Rudolph et al. [22] reveal in both strata slightly higher average numbers of CD8+ cells than those of CD4+ cells, marked differences in the ratio between CD4+ and CD8+ T cells exist between individual mares [18,22]. Intraepithelial lymphocytes mostly represent CD8+ lymphocytes [18,22]. CD8 is a marker of cytotoxic T cells, whereas CD4+ T cells include T-helper cells and regulatory T cells [18,22,56]. In the SC and SP, B-cells are reported as rare [56] or absent [22]. Cell counts of macrophages and plasma cells in the SC and SP range from 0 to 5 and from 0 to 3 per five 40× HPFs, respectively [22]. Schoon et al. [2] observe lymphocytes, plasma cells, and macrophages mainly during diestrus and glandular involution, whereas Watson and Thomson [56] detect no statistically significant difference in the numbers of CD4+ cells, CD8+ cells, and B cells in biopsies collected during estrus and diestrus. Plasma cells are mostly immunopositive for immunoglobulin (Ig) A [57]. Macrophages with intracytoplasmic hemosiderin-pigment (siderocytes) suggest previous parturition and persist 1–7 months postpartum [2,33]. Mild to moderate numbers of GE cells are immunopositive for IgA and IgM [57].

## 3. Innate Immune Defenses 

**Figure 3:**
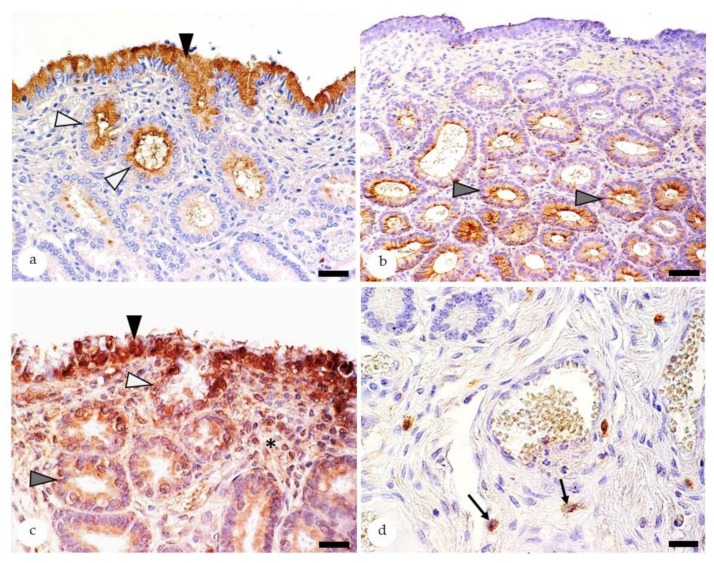
Equine endometria immunostaining for β-defensin (**a**), indoleamine 2,3-dioxygenase 1 (IDO 1) (**b**) and Toll-like receptor 4 (TLR 4) (**c**,**d**). Positive staining is shown by a brown color reaction; 3,3′ diaminobenzidin is used as chromogen. (**a**) In the healthy endometrium, β-defensin is mainly expressed in the cytoplasm of epithelial cells lining the luminal surface (black arrowhead) and glandular ducts (white arrowheads). Bar = 30 µm; (**b**) IDO 1 immunostaining is predominantly observed in glandular epithelial cells (gray arrowheads). Bar = 50 µm; (**c**–**d**) Staining for TLR 4 markedly differs between individual mares. Positive cells include epithelial cells of the luminal surface (**c**: black arrowhead), glandular ducts (**c**: white arrowhead), secretory portions of glands (**c**: gray arrowhead), stromal cells (**c**: asterisk), and/or mast cells (**d**: black arrows). Bars = 20 µm.

Components of the innate immunity include the antimicrobial peptide β-defensin [17,21], pattern recognition receptors such as Toll-like receptors [23,24] and Nucleotide-binding and oligomerization domain (NOD) receptors [58], the immunomodulatory enzyme indoleamine 2,3- dioxygenase 1 (IDO 1) [25], and proinflammatory cytokines [19,59,60]. The immunostaining patterns for β-defensin and IDO 1 in non-endometrotic areas and Toll-like receptor 4 (TLR 4) in different cell populations [23,24] are depicted in Figure 3. 

Endometrial biopsies immunostained for β-defensin; IDO 1; and TLRs 2, 4, and 6 (21–29 per study) are obtained from mares of varying ages, and they were collected during different stages of the endometrial cycle [17,21,23,24,25]. Only some biopsies are devoid of any alterations (2–3 per study), whereas the remaining have one to several endometrial diseases [17,21,23,24,25]. 

Immunostaining for β-defensin is mainly observed in LE cells and glandular ducts (GD), whereas epithelial cells of endometrial glands are rarely positive [17,21]. The luminal epithelium contains always positive cells [17,21] and 71–77% of examined biopsies display staining of GD [17,21]. In comparison, labelling of healthy mid and basal glands is observed in 23% and 4% of investigated biopsies, respectively [21]. The immunoreaction of LE cells and GD is predominantly located in the cytoplasm [17,21]. In comparison, GE cells have mostly a nuclear staining [17,21]. Rarely, β-defensin is detected in endometrial stromal cells and vascular smooth muscle cells [17]. 

In biopsies stained for IDO 1, healthy mid- and basal glands contain markedly higher numbers of positive epithelial cells than luminal epithelium and GD [25]. Macrophages are IDO 1 immunoreactive as well [25]. The staining is always located in the cytoplasm [25]. Notably, in a few mares (4 of 25 mares), IDO 1 expression in endometrial epithelial cells is reduced to absent whereas IDO 1 immunostaining in immune cells is preserved [25]. Genetic factors could be responsible for the markedly reduced epithelial IDO 1 expression in the endometrium of some mares [25].

Based on these investigations, there is no clear evidence for an influence of the stage of the endometrial cycle, inflammation, or angiosclerosis on immunoreaction for β-defensin and IDO 1 in endometrial epithelial cells [17,21,25]. In the majority of biopsies with endometrosis; however, healthy and endometrotic glands differ in their labelling for β-defensin and IDO 1 [21,25]. 

TLRs 2, 4, and 6 are found in epithelial cells and stromal cells and more rarely in vascular endothelia and smooth muscle cells, immune cells, and mast cells [23,24]. Epithelial cells show mainly a cytoplasmic staining and less commonly a nuclear immunoreaction, whereas all other cell populations display cytoplasmic labelling [23,24]. The expression patterns of TLRs 2, 4, and 6 within the different cell populations are highly variable between individual mares [23,24]. Further investigations are needed to reveal the factors that modulate the expression of TLRs 2, 4, and 6 in the equine endometrium; a contribution of genetic polymorphisms and/or individual differences in the exposure to pathogens or antigenic agents has to be considered [23].

## 4. The Endometrial Biopsy as Diagnostic Tool 

In case there are no clinical findings, the collection of one endometrial biopsy with a minimal size of 10 × 3 × 3 mm is considered as representative for the entire endometrium (Figure 1) [1,2]. As collection site, the implantation site, i.e., the junction between the uterine body and one uterine horn, is recommended [33,34]. 

Immediately after its collection, the biopsy should be placed in fixative. Nowadays, the routinely applied fixative is 10% buffered formalin, since it is suitable for histopathological and several immunohistological investigations. Subclassification of lymphocyte populations in fixed tissue samples, however, has so far not been archived on formalin-fixed tissue sections. For this purpose, the zinc-salt fixation is recommended [18,22]. Formalin- and zinc-salt fixed tissues can be used for embedding in paraplast blocks, sectioning and staining with hematoxylin-eosin (HE) and special stains to highlight particular tissue structures [18,22]. The microscopic evaluation of HE-stained sections will allow the diagnosis of endometrial diseases (Table 1) and prognosis of fertility (Table 2) [1,2,3]. The picrosirius red stain is an excellent tool for visualization of elastic fibers, collagen, as well as acid carbohydrates [61]. 

## 5. Endometrial Diseases 

The main endometrial diseases of mares as well as their subgroups are listed in Table 1. 

### 5.1. Equine Endometrosis 

Equine endometrosis is a frequent fertility-reducing disease of the equine endometrium. As defined by Schoon et al. [3], it is characterized by periglandular fibrosis associated with dysfunction of affected glandular epithelial cells. In previous publications, the terminology “periglandular fibrosis” was applied to describe this disease condition [1,2]. The severity and frequency of endometrosis rises with increasing age of the mare and a longer duration of barrenness [3,7,62]. The retrospective study of Ebert et al. [62] on 9120 biopsies submitted for routine diagnostic evaluation show the following age dependency of endometrosis: 32% in ≤5 year old mares, 66% in 6–10 year old mares, 84% in mares with an age from 11–15 years, 90% in mares aging 16–20 years, and 92.5% in >20 year old mares. Equine endometrosis has to be distinguished from endometriosis, a disease that occurs in women and menstruating primates and that is characterized by the implantation of dispersed endometrial tissue within the pelvic or abdominal cavities [63].

#### 5.1.1. Diagnostic Features of Endometrosis 

**Figure 4:**
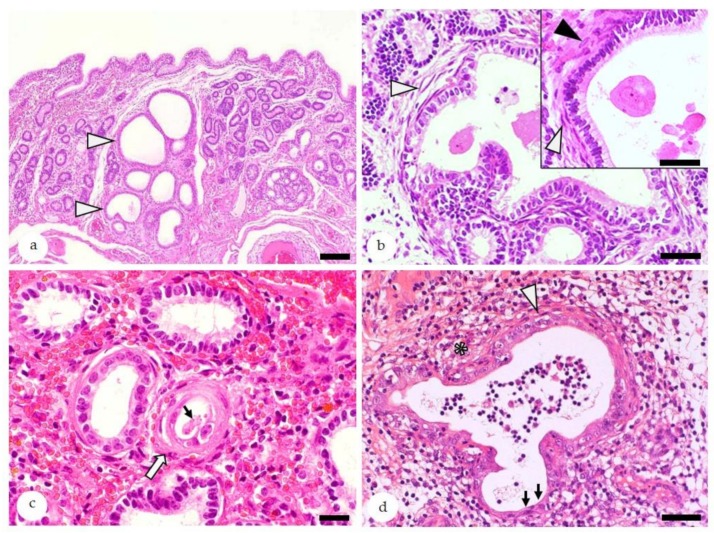
Equine endometrium, endometrosis, microscopic features, hematoxylin-eosin stain: (**a**) At low magnification, endometrotic glands (arrowheads) are recognized by periglandular fibrosis that is often associated with their cystic dilation, irregular shape, and/or nesting. Bar = 100 µm. (**b**) Nested endometrotic glands surrounded by inactive (white arrowheads) and active fibrosis (black arrowhead, inset). Inactive stromal cells have elongated hyperchromatic nuclei (white arrowheads), whereas activated stromal cells show oval hypochromatic nuclei (black arrowheads). Bar = 40 µm. Bar inset = 20 µm. (**c**) Mild inactive destructive endometrosis of a single gland (white arrow): In the destructive form, lining epithelia show degeneration/necrosis and intraluminal desquamation (thin arrow). Bar = 20 µm. (**d**) Concurrent presence of destructive endometrosis and endometritis: Periglandular fibrosis is labelled by a white arrowhead, and degeneration and attenuation of lining epithelia are labelled by black arrows, and inflammatory cells within the stroma are labelled by an asterisk. Bar = 50 µm.

The microscopic hallmark of endometrosis is the concentric arrangement of stromal cells and/or collagen fibers around affected glands (Figure 4) [3]. 

Endometrial biopsies with endometrosis contain diseased and healthy glands [28,64,65]. The degree of periglandular fibrosis (mild, moderate, or severe) is determined according to the number of periglandular layers of stromal cells and the number of fibrotic nests of glands within a linear microscopic field of view with a size of 5 mm [1,2,3]. 

Endometrosis can affect a single gland or multiple glands (“nested endometrosis”). In the nondestructive form of endometrosis, glandular epithelial cells are intact whereas their degeneration and necrosis are features of destructive endometrosis [3,8,64,65]. Periglandular stromal cells can show a metabolically active or an inactive differentiation [3,8,64,65]. Active stromal cells are characterized by an oval shape, pale cytoplasm, and ovoid hypochromatic nuclei, whereas inactive stromal cells are spindle-shaped with elongate hyperchromatic nuclei (Figure 4) [3,8,64,65].

The endometrial biopsy facilitates research on molecular features of endometrosis, since it allows the direct comparison of healthy and diseased glands within the same biopsy of an individual mare [28,64,65]. 

#### 5.1.2. Molecular Characteristics of Endometrosis 

In contrast to healthy glands, endometrotic glands often display abnormal immunostaining for cellular differentiation markers [16,28], alterations of the basement membrane [64], as well as a cycle asynchronous differentiation [64,65]. Fibrotic glands frequently acquire epithelial immunostaining for calponin [28] and vimentin [16,28,64], suggesting a myoepithelial differentiation. Stromal cells of endometrotic glands show decreased expression of calponin [28] and increased labelling for vimentin [16,28,64], desmin [16,27,28,64], and/or SMA [16,27,28,64]. The basement membrane can be thickened and/or discontinuous [64]. Cycle asynchronous differentiation is evidenced by aberrant expression of ESRα and PGR as well as secretory proteins including uteroglobin, uterocalin, calbindin, and glycogen [50,51,64,65,66]. 

The glandular epithelium of endometrotic glands also differ in their expression of β-defensin and IDO 1 as components of the innate immunity [21,25]. Healthy endometrial glands are mostly devoid of cytoplasmic labeling for β-defensin, and those with endometrosis frequently show cytoplasmic β-defensin expression [21,26]. Examining 26 biopsies with healthy glands and 19 biopsies with endometrotic glands, Schöniger et al. [21] observe cytoplasmic β-defensin labeling in healthy glands of one biopsy and in endometrotic glands of 15 biopsies. Most mares display a strong IDO 1 expression in healthy endometrial glands and a marked reduction or complete loss of IDO 1 immunoreaction in endometrotic glands [25,26] Only in a few mares, IDO 1 expression is very low to absent in epithelial cells of healthy and endometrotic glands [25,26].

#### 5.1.3. The Pathogenesis of Endometrosis—Still to be Revealed 

The initiating event as well as details about the pathogenesis of equine endometrosis are the focus of ongoing research [67,68]. 

Periglandular-accentuated mononuclear cell infiltrates (PAMC) have been proposed as a possible triggering event [20]. A mutual influence of endometritis and endometrosis has been hypothesized by several authors. Neutrophils attracted by bacterial infections release DNA components that lead to the formation of neutrophilic extracellular traps (NETs) [19,67,69]. In vitro studies show that the NETs components myeloperoxidase, elastase, and cathepsin G increase transcript levels of collagen type I and collagen type III as well as of factors that promote fibrosis, i.e., tumor growth factor β1 and tissue inhibitor of metalloproteinases depending on the stage of the estrous cycle and preexisting category of the endometrial tissue sample [19,67]. In addition, they decrease prostaglandin E2 that exerts an antifibrotic response after binding to the E prostanoid receptor [19]. Although these data suggest that acute inflammation may facilitate endometrosis, experimentally induced bacterial endometritis with subsequent treatment (20 mares) was not associated with progression of endometrosis over a 2-year observation period in 90% of the mares [64]. Higher categories (Table 2) in endometrial biopsies of susceptible mares [60,70] support the assumption that preexisting endometrosis predisposes to endometritis. This assumption is further supported by an increasing incidence of endometritis in older mares [62,71]. 

The highly significant correlation between presence and severity of endometrosis and angiosis [4] indicates that both diseases influence each other. It may be assumed that perfusion disorders facilitate progression of endometrosis.

### 5.2. Endometritis—Subclassification and Disease Susceptibility 

The incidence of endometritis in biopsies submitted for routine histological examination ranges from 29–44% [62]. The histologically determined frequency of endometritis is likely lower than its “true” incidence, since acute suppurative endometritis is mostly diagnosed solely by clinical and/or cytological examination [12]. Ebert et al. [62] reveal an age-related increasing frequency of endometritis in equine endometrial biopsies (n = 9120), i.e., 29% in ≤ 5-year-old mares, 40.5% in 11–15-year-old mares, 42.5% in mares aging 16–20 years, and 44% in >20-year-old mares.

**Figure 5:**
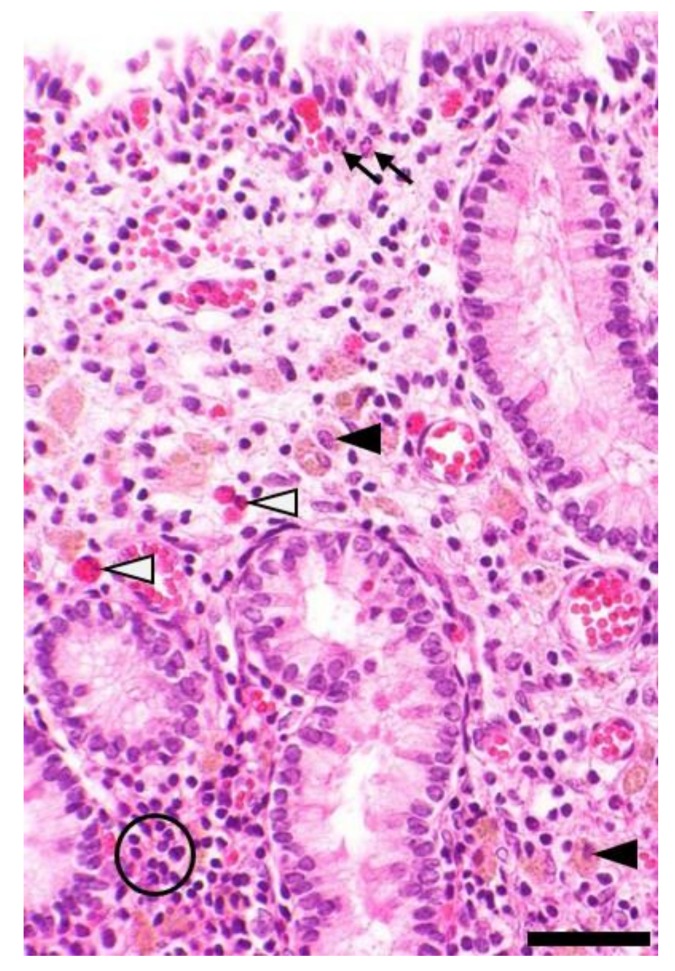
Equine endometrium, microscopic findings in endometritis, hematoxylin-eosin stain: Depicted is the presence of moderate subacute suppurative endometritis characterized by stromal infiltration with mainly lymphocytes (circle) mixed with a few neutrophils (thin arrows). There are also moderate numbers of siderocytes (black arrowheads) indicative of previous hemorrhage and a few eosinophils (white arrowheads). Bar = 50 µm.

Microscopically, endometritis is defined as presence of inflammatory and immune cells within the endometrium that exceed the physiological norm according to the stage of the endometrial cycle (Figure 5) [2,3,36]. Endometritis is diagnosed as superficial if the SC is affected and as deep if it is (also) present within the SP [17].

#### 5.2.1. Suppurative Endometritis 

Suppurative endometritis is mostly caused by an ascending bacterial infection [72]. In rare cases, it may be evoked by activation of dormant bacteria [73]. The physiologically observed transient post-breeding endometritis also manifests as suppurative inflammation [2,3]. 

A transient endometritis after mating or insemination is a physiological reaction and has the function to eliminate excess spermatozoa, seminal plasma, cellular debris, and bacteria [12]. Similar to after bacterial infection, the inflammatory response is characterized by the production of proinflammatory cytokines, i.e., interleukin (IL) 1β, IL8, and IL6 and tumor necrosis factor-α within 2 h [60]; recruitment of neutrophils within 30 min to 2 h [10,11,74]; and an influx of T cells within 6 h [75]. Neutrophils release prostaglandin F2α, resulting in myometrial contractions to remove intrauterine inflammatory contents [11,74]. In addition, anti-inflammatory cytokines, i.e., IL10 and IL1RN, increase within 6 h [60,74]. The inflammation is usually cleared within 48–72 h [3,10]. 

#### 5.2.2. Nonsuppurative Endometritis 

Nonsuppurative endometritis is characterized by the infiltration of the endometrium with lymphocytes that are often accompanied by plasma cells, while B-cells and macrophages are uncommon [22]. T cells are increased in the luminal and glandular epithelium, SC, and SP [18,22]. In endometria of 24 mares with a superficial mild nonsuppurative endometritis, numbers of CD3+ lymphocytes and plasma cells within the SC range from 129 to 479 and from 2 to 115 per five 40× HPFs, respectively [22]. 

Intraepithelial T cells are predominantly CD8+ T cells [18,22]. Although, on average, stromal CD8+ T cells are also more frequent than stromal CD4+ T cell, individual mares display marked variations in the ratio between both T cell populations [22]. There is evidence for a contribution of CD3+ CD4-/CD8- T cells as well [22]. 

The cause and pathogenesis of nonsuppurative endometritis is still undetermined. The marked variations in stromal CD4+ and CD8+ T cells between individual mares [18,22] indicate the existence of subtypes of nonsuppurative endometritis, possibly related to different etiologies or variations in resident immune cell populations between individual mares [18,22].

#### 5.2.3. Endometritis Eosinophilica 

Endometritis eosinophilica (EE) can be subclassified according to the distribution of eosinophils within the equine endometrium [54]. Focal EE is characterized by ≥40 eosinophils within one 40× HPF, whereas in multifocal EE, two or more HPFs contain ≥20 eosinophils [54]. The diagnostic hallmark of diffuse EE is a mean value of ≥15 eosinophils per 40× HPF [54]. Multifocal to confluent EE is diagnosed if at least two 40× HPFs contain ≥ 40 eosinophils and a mean value of ≥15 eosinophils per 40× HPF is reached [54]. 

Eosinophilic infiltration/inflammation can be evoked by infection with certain bacteria, virus, fungi, and parasites as well as by allergic reactions [54]. In the endometrium, pneumovagina [76] and progestin application [77] are additional possible etiologic factors. The underlying cause of EE, however, often remains undetermined. In these cases, EE may have to be considered as an endometrial manifestation of an idiopathic eosinophilic inflammatory condition [54]. 

#### 5.2.4. Granulomatous Endometritis 

Reported cases of granulomatous endometritis are rare and include pyogranulomatous reactions with intralesional bacteria, xanthogranulomatous endometritis, and granulomatous inflammation with contribution of numerous eosinophils [78]. 

#### 5.2.5. Cytology as Diagnostic Tool for Endometritis

The detection of elevated numbers of neutrophils, eosinophils, and/or lymphocytes in uterine lavage, cytological smears, or scrapings confirms endometritis [12]. A negative result, however, does not rule out endometritis, since these methods only detect inflammatory cells that are present within the uterine lumen (cytological smears and scrapings) and the superficial mucosa (scrapings). In addition, these examinations cannot precisely predict the subtype of endometritis as well as its severity and distribution within the lamina propria mucosae (superficial versus deep).

#### 5.2.6. The Susceptible Mare

Susceptibility of a mares to persisting endometritis is characterized by a failure to resolve (experimental) bacterial or breeding-induced endometritis within 72 h [3,59,74]. The reported incidence in broodmares is about 15% [74], and an older age and endometrosis represent predisposing factors [74]. Other associated factors are anatomical abnormalities of uterus and cervix [12], an increased angle of declination of the vulva [12], impaired myometrial contractions [11,74], and a dysregulation of inflammatory and immune mechanisms [12,59,60,74,79]. Elevated intrauterine nitric oxide levels [70], a prolonged proinflammatory response [59], and reduced levels of anti-inflammatory cytokines [60] have been detected in susceptible mares. 

### 5.3. Endometrial Maldifferentiation During the Breeding Season 

Endometrial maldifferentiation only represents a pathological condition during the breeding season. It includes glandular inactivity [13] as well as unequal [5,6,80] and irregular glandular differentiations [5,6,80]. Over the entire year, irregular and unequal differentiation occurs with a frequency of 13–17% (n = 9120 biopsies) [62], with its incidence during the breeding season even lower. A much higher frequency of glandular maldifferentiation during the breeding season, however, is diagnosed in retired sport mares [13]. 

#### 5.3.1. Glandular Inactivity During the Breeding Season 

Glandular inactivity is characterized by the presence of small glands that have narrow lumina and are lined by flattened epithelial cells [13]. The expression of ESR and PGR in glandular epithelial cells and stromal cells is very weak [15], and stromal cells may stain for desmin [15,16]. In retired sport mares, the incidence of glandular inactivity during the breeding season is about 8% [13]. 

#### 5.3.2. Irregular Glandular Differentiation During the Breeding Season

**Figure 6:**
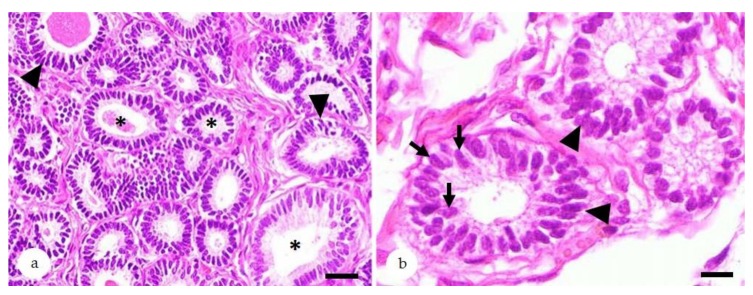
Equine endometrium, glandular maldifferentiation, hematoxylin-eosin stain: (**a**,**b**) Biopsies with irregular proliferative differentiation. Endometrial glands vary in size and shape (**a**: asterisks). Glandular epithelial cells show segmental to circumferential pseudostratification (**a**,**b**: arrowheads). They are lined by epithelia containing ovoid or elongate nuclei with loss of polarity that are located at random within the cytoplasm (b: arrows). Bar (a) = 40 µm; Bar (b) = 10 µm.

Irregular glandular differentiation can manifest as irregular secretory, irregular proliferative (Figure 6), or complete irregular differentiation [5,6]. The former two forms show a retained predominant secretory or proliferative morphology [5,6]. If no residual functional state can be recognized, complete irregular differentiation is diagnosed [5]. The morphological findings are accompanied by an altered immunostaining for ESRα and PGR [5,13,81], intermediate filaments [5,80,81], scretory proteins [82], and laminin as component of the basement membrane [6,80]. 

The underlying cause is a hormonal dysfunction, e.g., caused by ovarian tumors [5,81], ovarian dystrophy [5], long-term progestin application [77], or athletic performance [13]. In the study of Kilgenstein et al. [13], 42% of retired sport mares display an irregular glandular differentiation. The disorder is more frequency observed in sport mares that retired ≤1 year than in those being retired for ≥2 years [13]. Cases without detectable underlying cause are diagnosed as idiopathic differentiation disorders [7]. Irregular glandular differentiation is potentially reversible. After ovariectomy [81] or termination of a long-term progestin application [82], resolution of the abnormal differentiation [81,82] and successful pregnancies [81] are reported. 

#### 5.3.3. Unequal Glandular Differentiation During the Breeding Season

Unequal glandular differentiation is characterized by focal or multifocal groups of glands with a cycle asynchronous differentiation [5,6]. In addition, an abnormal expression of hormone receptors [5,6,80] and secretory proteins [82] and often the presence of a discontinuous basement membrane [6] are observed. In addition, the stroma in areas with UGD contained slightly higher numbers of SMA- and desmin-positive stromal cells than the stroma of healthy endometrial areas [27]. UGD is considered a sequel to partial hormonal unresponsiveness of affected areas [5,6,80], likely caused by initial stages of endometrosis in adjacent tissue areas [27]. 

### 5.4. Angiopathies

Angiopathies of the equine endometrium can be subdivided into the less common inflammatory lesions (perivasculitis and vasculitis) and the frequent degenerative angiopathies (angioses, synonym: angiosclerosis) [83].

#### 5.4.1. Angiosis—Diagnostic Features

**Figure 7:**
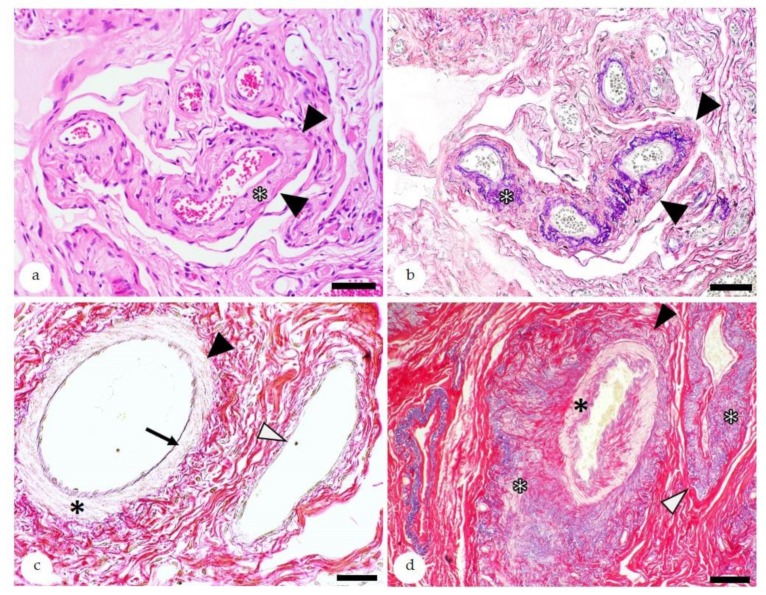
Histopathology of the equine endometrium depicting angiosis: (**a**) Medium-sized vessels with moderate angiosclerosis (black arrowheads). The diagnostic hallmark on hematoxylin-eosin (HE)-stained sections is the loss of cellular detail in the vessel wall and the deposition of eosinophilic extracellular matrix (asterisk). Bar = 50 µm. (**b**) Serial section of the vessels observed in “a” (black arrowheads) stained with the picrosirius red stain to highlight collagen (red) and elastic fibers (lila to black). The special stain confirms the presence of elastofibrosis due to the increased deposition of collagen and elastic fibers within the vessel wall (asterisk). Bar = 50 µm; (**c**) Normal artery (black arrowhead) and vein (white arrowhead) stained with picrosirius red: The artery contains a thin continuous lamina elastica interna (arrow) and a muscular tunica media (asterisk). In comparison to the artery, the vein has a thin tunica media. Bar = 40 µm; (**d**) Picrosirius red stain of an artery (black arrowhead) and vein (white arrowhead) with marked fibroelastosis: In the artery, the intima and media are mildly to moderately effaced by the deposition of elastic fibers and collagen (black asterisk). The adventitia is markedly expanded by the presence of elastic fibrers and collagen (white asterisk). The vein shows marked elastofibrosis of the adventitia (white asterisks), whereas the intima and media are mainly unaffected.

Angioses are characterized by an increased deposition of fibrous connective tissue and/or elastic fibers within the wall of endometrial vessels (Figure 7) [4,7,83]. They can be further classified according to the type of affected vessel (artery or vein); the affected vascular layer(s); and the quality of the alteration, i.e., elastosis, fibrosis, fibroelastosis, elastofibrosis, as well as its severity (mild, moderate, or severe) [4]. Although vascular alterations can already be diagnosed in HE-stained sections, for a concise characterization of alterations, the picrosirius red stain is an excellent method (Figure 7) [71,83,84,85]. 

Vascular alterations in endometrial vessels are associated with similar vascular lesions in the uterine artery [7,84] and ovarian vessels [85]. Severe sclerosis of endometrial arteries provides a risk factor for rupture of the uterine artery that is usually fatal [7,84]. 

#### 5.4.2. Angiosis—Influence of Age and Parity 

The incidence of angioses and their severity increase in older mares with multiple foalings, whereas age and parity represent independent predisposing factors [4,7,62,83]. A retrospective study on biopsies (n = 9120) submitted for routine investigation shows the following age influence on the incidence of angiosis, i.e., 18% in ≤5 year old mares, 44% in 6–10 year old mares, 62% in mares with an age from 11–15 years, 72% in 16–20 year old mares, and 82% in those >20 years old [62]. Unaffected vessels are only seen in endometrial biopsies of younger maiden mares [4]. Older maiden mares often show mild changes in the intima and adventitia of endometrial vessels [4,83], whereas the media is commonly unaffected [83]. Pregnancy-related changes usually affect all layers of the vessel wall [4] and are comparable to “pregnancy-sclerosis” of other species [4,83]. They are characterized by disruption, lamellation, and thickening of the membrana elastica interna; atrophy of smooth muscle cells in the tunica media; elastosis and fibrosis of the intima; media and adventitia; and/or calcification within the vessel wall [4,83] and likely result from vascular remodeling during pregnancy [83]. 

### 5.5. Endometrial Cancer: A Rare Disease in Mares

In contrast to women, endometrial tumors are rarely reported in mares of all age groups [33,86]. To the authors knowledge, no published cases of uterine adenoma exist. Publications on endometrial adenocarcinomas are restricted to single case reports [86,87,88,89,90]. Endometrial adenocarcinomas are reported in an 11-year-old Arabian horse [88], a 14-year-old mare [87], a 16-year-old Quarter horse [90], and a 25-year-old Przewalski mare [89]. Mares were either nulliparous [89] or had delivered one [88] to several foals [90]. Tumors showed papillary [87], acinar and tubular [87,88,89,90], and solid [89] growth patterns, with invasion of the myometrium [87,88,89,90], extension in the cervix [90], and intralymphatic [87,89,90] and intravascular tumor cell emboli [90]. Metastases to regional lymph nodes [87,89] as well as distant metastases are reported [87,88,90]. The latter affected lungs [87,88], trachealbonchial lymph nodes [87], liver [88], spleen [88], mesentery [88], omentum [88], serosal surfaces of intestines [88], urinary bladder [90], ureter [90], ovaries [90], adrenal glands [90], and bone marrow [90]. 

In addition, an endometrial fibrosarcoma with invasion in the myometrium; tumor cell emboli in lymphatics; and metastases in lungs, liver, and spleen is described in an 18-year-old Warmblood mare [91]. Other published neoplasms with involvement of the endometrium are multicentric lymphosarcomas, i.e., T-cell lymphosarcoma in a 22-year-old pony mare [92] as well as B-cell lymphosarcomas in a 5-year-old Quarter horse mare [93] and an 8-year-old Thoroughbred mare [94].

Endometrial stromal polyps that represent tumor-like lesions have been diagnosed in a 1-year-old thoroughbred filly [95] and a 2-year-old Arabian cross filly [96].

## 6. Discussion

### 6.1. Different Equine Endometrial Diseases: A Common Cause for Subfertility?

The equine conceptus arrives in the uterine lumen at day 6 after ovulation, and the preimplantation period lasts until day 17 of pregnancy [97]. During this time, the equine conceptus moves within the uterine cavity and is solely nourished by uterine milk [97]. The equine placenta is microcotyledonary and epitheliochorial [97]. After established placentation, in addition to vascular supply of nutrients, the fetus is still nourished by uterine milk [97]. 

The presence of endometrial diseases, i.e., glandular maldifferentiation, endometrosis, and angiosis, impair production of uterine milk as well as placentation [7,97]. In addition, the area of placental exchange is also reduced by cellular infiltrates [97]. This predisposes to fetal malnutrition, fetal death, and abortion [65,66,82,97].

The altered expression of components of the uterine milk in diseased glands [65,66,82] is likely a sequel to the disease-associated abnormal expression of ESRα, PGR, and cytoskeletal proteins together with alterations in the basement membrane [6,64,65,80]. Moderate to severe arterial lesions can reduce vascular perfusion of the uterus [4,7] and can thus impair the nutrient supply of the equine fetus [97]. Vascular lesions in the ovarian arteries predispose to functional ovarian alterations [85] and subsequent glandular endometrial maldifferentiation with the possible consequence of malnutrition of the conceptus [82]. 

In addition, uterine inflammation at the time of breeding or insemination reduces viability and motility of spermatozoa and lowers pregnancy rates [98,99,100]. Underlying causes are binding of spermatozoa to neutrophils that facilitates their phagocytosis and aggregation [98,99] as well as damage of spermatozoa due to the presence of inflammatory mediators [100].

### 6.2. Molecular Features of Equine Endometrial Pathophysiology—an Important Research Aspect

So far, the focus of molecular studies on the equine endometrium was to reveal the molecular mechanisms involved in physiological aspects and the pathogenesis of endometrial diseases and associated subfertility of mares [5,15,16,17,18,19,20,21,22,23,24,25,26,27,28,40,51,64,65,66,80,81,82]. Some of the described molecular markers qualify as biomarkers for measurement of regular and abnormal cellular function within the equine endometrium.

#### 6.2.1. Biomarkers for Equine Endometrial Health and Disease 

Molecular markers that specifically recognize endometrial components with different forms of cellular alterations are listed in Table 3. Quantification of the protein expression of these markers and their direct visualization within the healthy and diseased tissue is also possible [5,6,13,16,17,21,25,27,28,40,51,64,65,66,80,81,82]. Thus, these molecules have the potential to serve as biomarkers for functional deviations of epithelial and stromal cells within the equine endometrium. Their evaluation in the context of tissue morphology will likely help to diagnose cellular dysfunctions associated with disease entities and their subtypes. The comparison of endometria with different disease subtypes as well as their combinations will likely reveal the degree of impact that is imposed by a specific disease state on a defined cellular dysfunction. As a future perspective, this could lead to an objective measurement of endometrial dysfunction and would likely help to refine the fertility prognosis for an individual mare. 

So far, the fertility prognosis is based on the categorization scheme of Kenney and Doig [1] (Table 1) together with the amendments of Schoon et al. [3,7]. The categorization [1] provides a fertility prognosis with a wide range, e.g., category IIb mares have a 10–50% change to conceive, to maintain pregnancy, and to deliver a live foal. The categorization scheme also does not include all factors with an influence on fertility [3,7]. Additional factors to be considered are irregular and unequal glandular differentiation during the breeding season [7], severe angiosis [3,7], age of the mare ≥18 years [7], and a worser prognosis of certain types of endometrosis, i.e., endometrosis of basal glands [3] and the destructive type [3,65]. 

#### 6.2.2. Molecular Research to Improve Treatment 

Investigation on disease-associated molecular features reveal additional insights into predisposing factors and likely mutual influences between endometrial diseases [18,20,22,27,28,64,66]. In addition, they assist in the development of prophylactic regimes and novel treatment options. Intrauterine infusions of mares with autologous conditioned serum [101], allogenic bone marrow-derived mesenchymal stem cells (MSCs) [101], or autologous endometrial MSCs [102] revealed the anti-inflammatory properties of these treatments. This suggest their possible prophylactic or therapeutic use in mares susceptible to persistent endometritis [101,102]. Due to the lack of available therapies, currently equine endometrosis is regarded as an irreversible disease [3,8]. However, it has been referred that transplantation of allogenic adipose tissue-derived MSCs in the endometrium of mares results in a partial to complete reversal of the altered expression of cytoskeletal proteins in epithelial and stromal cells of endometrotic areas [14]. Thus, stem cell therapy may provide a suitable treatment for endometrosis as well [14]. Further work needs to be done to establish the clinical applicability of research data on possible novel treatment options for persistent endometritis or endometrosis.

## Figures and Tables

**Table 1:** Classification of endometrial diseases in mares.

Disease Entities	Subgroups
Endometrosis	Single Glands/Nested Glands/Basal Glands Active/Inactive/Mixed Nondestructive/Destructive
Endometritis	Superficial/Deep Suppurative/Nonsuppurative/Endometritis/ Eosinophilica/Granulomatous
Maldifferentiation during the breeding season	Inactive Irregular Secretory/Irregular Proliferative/Completely Irregular Unequal
Angiosis	Arterial/Venous Intima/Media/Adventitia Fibrosis/Elastosis/Elastofibrosis/Fibroelastosis
Neoplasia	Adenoma/Adenocarcinoma

**Table 2:** Categorization scheme of Kenney and Doig [1] modified by Schoon et al. [2,3].

Category	Microscopic Findings and Additional Factors	Estimated Foaling Rate *
**I**	No significant alterations	>80–90%
**IIa**	Mild endometritis OR Mild endometrosis OR Mild lymphatic lacunae OR Partial endometrial atrophy during the late breeding season	50–80%
**IIb**	Moderate endometritis OR Moderate endometrosis OR Moderate lymphatic lacunae OR Barrenness ≥2 years OR Combined presence of two category IIa findings	10–50%
**III**	Marked endometritis OR Marked endometrosis OR Marked lymphatic lacunae OR Deep endometrial atrophy during the breeding season OR Combined presence of ≥3 category IIa or ≥2 category IIb or III findings	<10%

* Term pregnancy and delivery of a live foal.

**Table 3:** Proposed biomarkers for regular and altered cellular functions in the equine endometrium.

Molecule	Cell(s)/Structure	Normal and Altered Functions
Estrogen Receptor	E, S	Hormonal Responsiveness
Progesterone Receptor	E, S	Hormonal Responsiveness
Vimentin	E, S	Cellular Differentiation
Desmin	S	Cellular Differentiation
Smooth muscle actin	S	Cellular Differentiation
Calponin	E, S	Cellular Differentiation
Laminin	BM	Interaction GE/S
Uteroglobin	E	Secretory activity
Uterocalin	E	Secretory activity
Uteroferrin	E	Secretory activity
Calbindin	E	Secretory activity
β-Defensin	E	Immune defense
Indoleamine 2,3 dioxygenase	E	Immune defense

E = epithelial cells; S = stromal cells; BM = basement membrane; GE = glandular epithelium.
